# Supplementary material for: The microbial metabolite p-Cresol induces autistic-like behaviors in mice by remodeling the gut microbiota
Source: Microbiome. 2021 Jul 8;9:157. doi: 10.1186/s40168-021-01103-z (PMC8268286; doi:10.1186/s40168-021-01103-z)
Supplement: Supplementary file 2 — Additional file 1: Fig. S1. p-Cresol treatment does not impact general physiological parameters, increases urinary and fecal p-Cresol excretion but not its serum levels. Fig. S2. p-Cresol treatment-induced behavioral alterations: additional parameters in the 3-chamber, dyadic social interactions and stereotypies tests and results from tests assessing locomotor activity, anxiety or cognition (relative to Fig. 1). Fig. S3. Autistic-like behaviors persist after discontinuation of p-Cresol for 4 weeks. Fig. S4. Additional behavioral parameters for the 3-chamber, dyadic social interactions, motor stereotypies tests and anxiety in FMTControl and FMTp-Cresol mice (relative to Fig. 4). Fig. S5. p-Cresol microbial biosynthetic pathways (relative to Fig. 5). Fig. S6. FMT experiments from control donors (FMTControl) to Control or p-Cresol-treated recipient mice: additional behavioral parameters in the 3-chamber, dyadic social interactions and stereotypies tests (relative to Fig. 6). Tab. S5: Recapitulation of the behavioral tests performed in the course of the study. [file 40168_2021_1103_MOESM2_ESM.pdf]

Additional File 1.

The microbial metabolite *p*-Cresol induces autistic-like behaviors in mice by remodeling the gut microbiota

Bermudez-Martin, P., Becker, J. A. J., Caramello, N., Fernandez, S. P., Costa-Campos, R., Canaguier, J., Barbosa, S., Martinez-Gili, L., Myridakis, A., Dumas, M.-E., Bruneau, A., Cherbuy, C., Langella, P., Callebert, J., Launay, J.-M., Chabry, J., Barik, J., Le Merrer, J., Glaichenhaus, N., Davidovic, L.

File content:

-Supplementary figures (6)

-Supplementary figures legends (6)

-Supplementary table 5 (Tab. S5)

-Supplementary tables legends (5)

SUPPLEMENTARY FIGURES

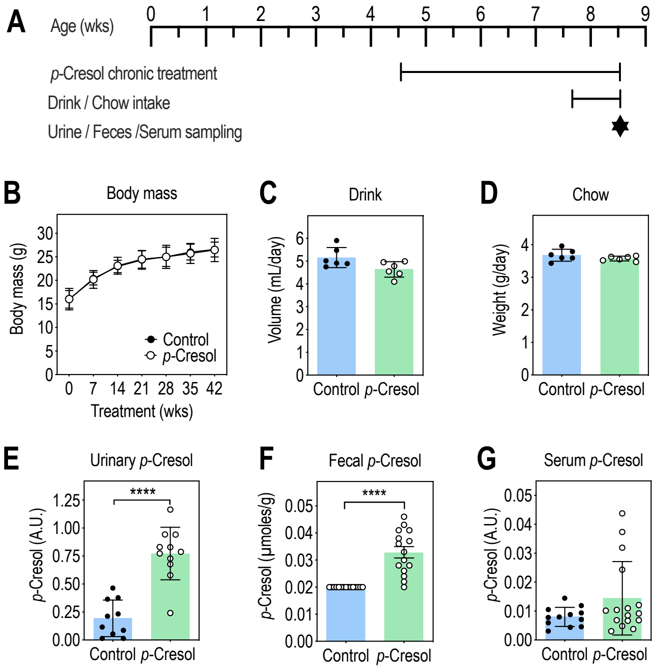

Fig. S1. *p*-Cresol treatment does not impact general physiological parameters, increases urinary and fecal *p*-Cresol excretion but not its serum levels

(A) Timeline of the experiment.

(B) Body weight follow-up (n=24/group); 2-way ANOVA: p(Treatment)=0.8884, p(Time)<0.0001, p(Treatment x Time)=0.9971; Šidák's post hoc tests for treatment effect: p>0.99. Note that curves from *p*-Cresol and Control animals overlap.

(C) Drink intake (n=6/group, each point representing the mean consumption per animal in one cage of 5 animals); Mann-Whitney U-test: p=0.0931.

(D) Food intake (n=6/group, each point representing the mean consumption per animal in one cage of 5 animals); Mann-Whitney U-test: p=0.3939.

(E) Urinary levels of *p*-Cresol after 4 weeks treatment (n=10 Control, n=11 *p*-Cresol); Mann-Whitney U-test: ****p<0.0001.

(F) Fecal levels of *p*-Cresol after 4 weeks treatment (n=15 Control, n=15 *p*-Cresol); Mann-Whitney U-test: ****p<0.0001.

(G) Serum levels of *p*-Cresol after 4 weeks treatment (n=12 Control, n=16 *p*-Cresol); Mann-Whitney U-test: p=0.3238.

(B) Data are presented as means ± SD.

(C-G) Data are presented as dot-plots featuring means ± SD.

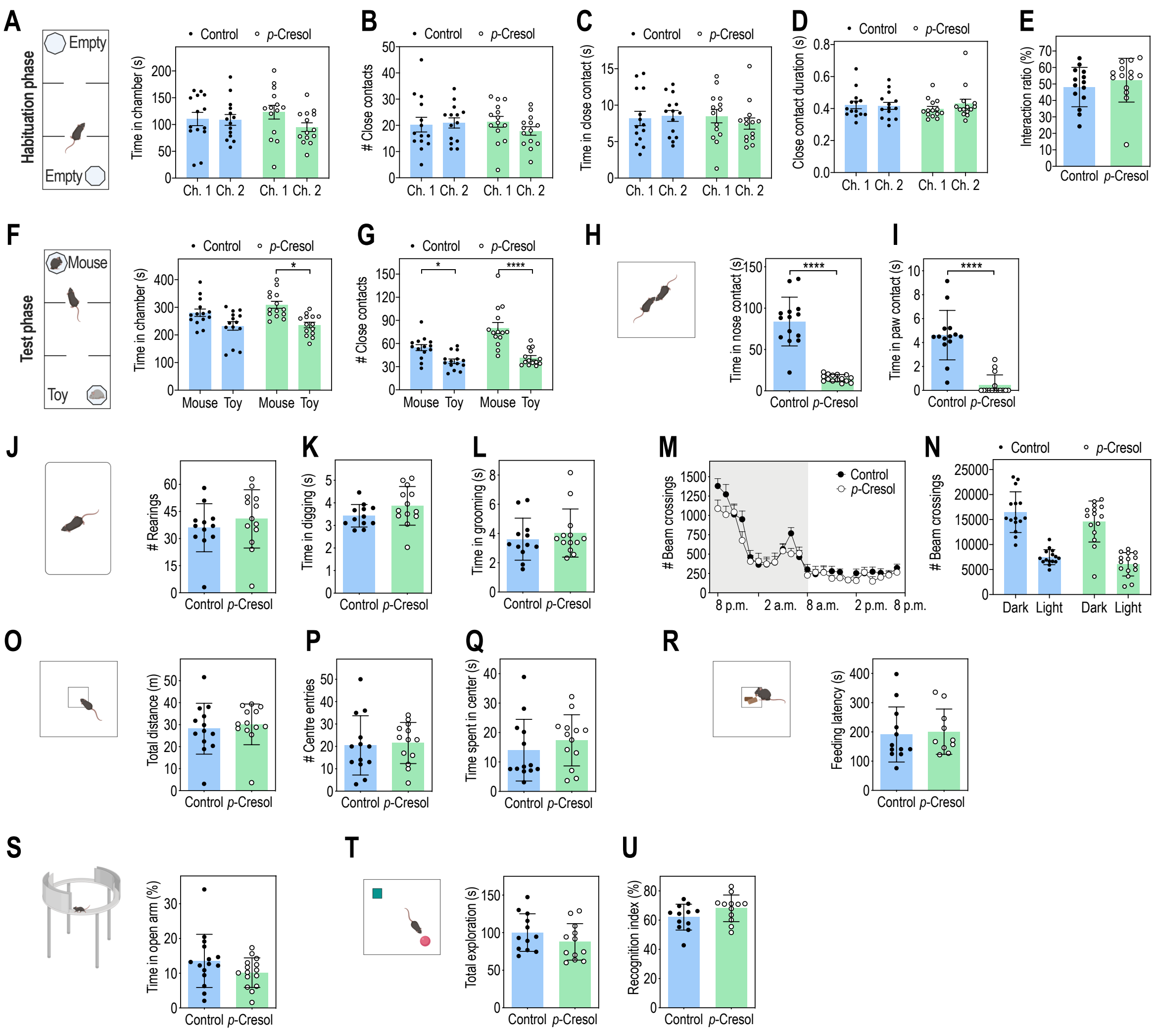

Fig. S2. *p*-Cresol treatment-induced behavioral alterations: additional parameters in the 3-chamber, dyadic social interactions and stereotypies tests and results from tests assessing locomotor activity, anxiety or cognition (relative to Fig. 1)

(A-E) Additional parameters during the habituation phase in the 3-chamber test (n=14/group):

(A) Time spent in each chamber; 2-way ANOVA: p(Treatment)=0.9498, p(Chamber)=0.3018, p(Treatment x Chamber)=0.3599; Šidák's post hoc tests for chamber effect: p>0.05.

(B) Number of close contacts with the empty wired cage in each chamber; 2-way ANOVA: p(Treatment)=0.6839, p(Chamber)=0.4390, p(Treatment x Chamber)=0.2648; Šidák's post hoc tests for chamber effect: p>0.05.

(C) Time spent in close contact with the empty cage in each chamber; 2-way ANOVA: p(Treatment)=0.7265, p(Chamber)=0.6260, p(Treatment x Chamber)=0.3188; Šidák's post hoc tests for chamber effect: p>0.05.

(D) Mean duration of each close contact with the empty cage in each chamber; 2-way ANOVA: p(Treatment)=0.8330, p(Chamber)=0.4995, p(Treatment x Chamber)=0.3378; Šidák's post hoc tests for chamber effect: p>0.05.

(E) Interaction ratio (percentage of time spent exploring the wire cage in Chamber 1 relative to the total time spent exploring both wire cages in Chamber 1 and 2); Mann-Whitney U-test: p=0.265.

(F, G) Additional parameters during the test phase in the 3-chamber test (n=14/group):

(F) Time spent in each chamber containing the mouse interactor or the toy mouse; 2-way ANOVA: p(Treatment)=0.0061, p(Preference)=0.0019, p(Treatment x Preference)=0.4797; Šidák's post hoc tests for Mouse vs. Toy preference effect: p>0.05 for Control group, * p<0.05 for *p*-Cresol group.

(G) Number of close contacts with the mouse interactor or the toy mouse; 2-way ANOVA: p(Treatment)=0.0035, p(Mouse-Toy)<0.0001, p(Treatment x Mouse-Toy)=0.0257; Šidák's post hoc tests for Mouse vs. Toy preference effect: *p<0.05, ****p<0.0001.

(H, I) Additional parameters in the dyadic social interaction test (n=14 Control, n=15 *p*-Cresol):

(H) Total time spent in nose and (I) paw contact; Mann-Whitney U-test: ****p<0.0001.

(J-L) Additional parameters in the motor stereotypies test (n=11 Control, n=12 *p*-Cresol):

(J) Number of rearings; Mann-Whitney U-test: p=0.3400.

(K) Time spent in digging; Mann-Whitney U-test: p=0.4613.

(L) Time spent in grooming; Mann-Whitney U-test: p=0.1366.

(M, N) Locomotor activity in actimetry chambers (n=15/group):

(M) Horizontal activity follow-up over 24 h; 2-way ANOVA: p(Treatment)=0.1057, p(Time)<0.0001, p(Treatment x Time)=0.3610; Šidák's post hoc tests for treatment effect: p>0.05.

(N) Cumulative activity in the dark or light phase; 2-way ANOVA: p(Treatment)=0.0909, p(Phase)<0.0001, p(Treatment x Phase)=0.7513; Šidák's post hoc tests for treatment effect: p>0.05.

(O-Q) Open-field test (n=13/group):

(O) Total distance travelled; Mann-Whitney U-test: p=0.3107.

(P) Number of centre entries; Mann-Whitney U-test: p=0.6047.

(Q) Time spent in the center of the area; Mann-Whitney U-test: p=0.3292.

(R) Novelty suppressed feeding (NSF) test (n=12 Control, n=10 *p*-Cresol): latency to first bite; Mann-Whitney U-test: p=0.5707.

(S) Zero-maze test (n=15/group): percentage of time spent in open arm; Mann-Whitney U-test: p=0.1485.

(T, U) Novel object recognition (NOR) test (n=12/group):

(T) Total time spent exploring the objects during the test phase; Mann-Whitney U-test: p=0.1978.

(U) Recognition index (percentage of time spent exploring the novel object relative to the total time spent exploring the old and new objects); Mann-Whitney U-test: p=0.1135.

(A-L, N-U) Data are presented as dot-plots featuring means ± SD.

(M) Data are shown as means ± SD.

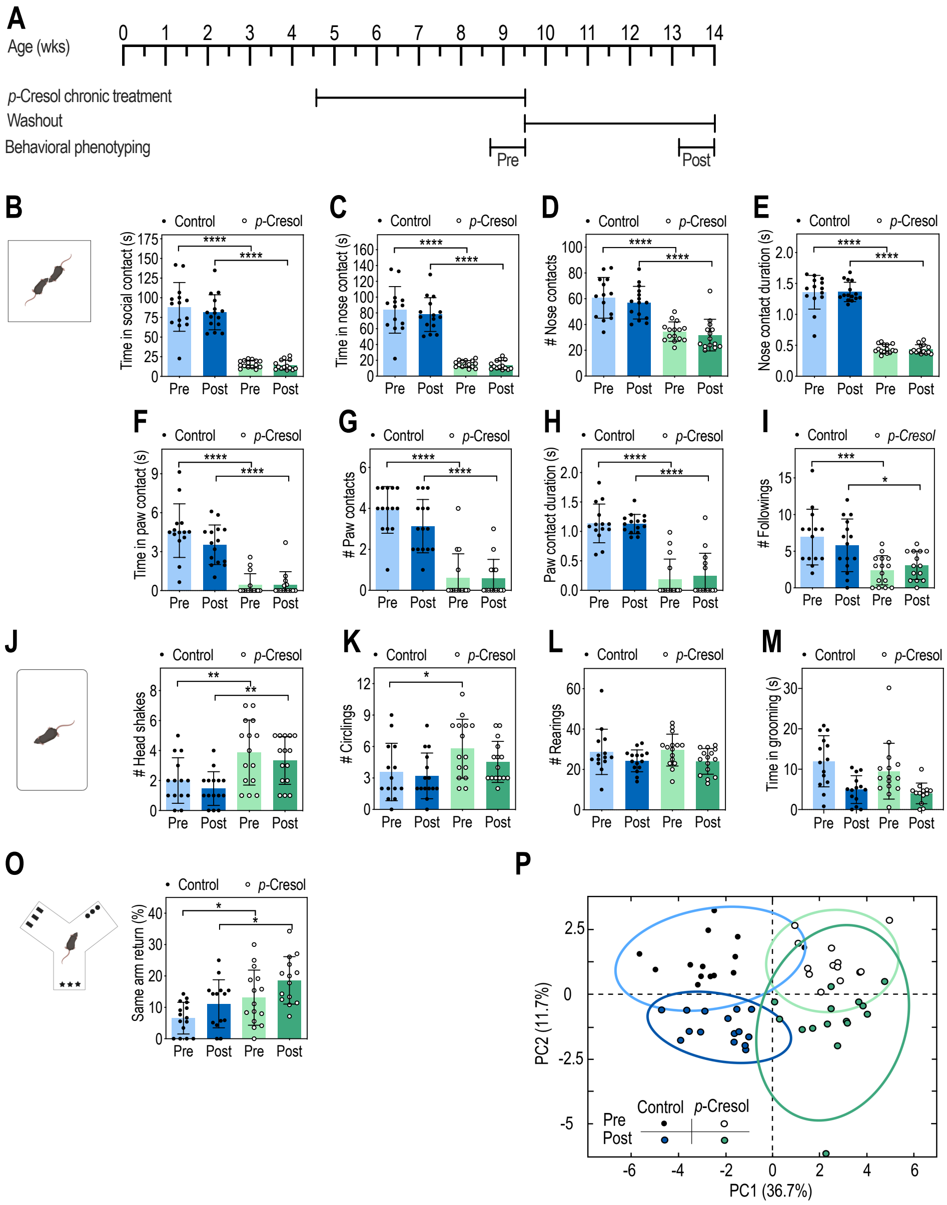

Fig. S3. Autistic-like behaviors persist after discontinuation of *p*-Cresol for 4 weeks

(A) Timeline of the experiment.

(B-I) Dyadic social interaction test (n=14 Control, n=15 *p*-Cresol):

(B) Total time spent in social contact; 2-way ANOVA: p(Treatment)<0.0001, p(Washout)=0.4194, p(Treatment x Washout)=0.5883; Šidák's post hoc tests for treatment effect: ****p<0.0001.

(C) Time spent in nose contact; 2-way ANOVA: p(Treatment)<0.0001, p(Washout)=0.4525, p(Treatment x Washout)=0.6450; Šidák's post hoc tests for treatment effect: ****p<0.0001.

(D) Number of nose contacts; 2-way ANOVA: p(Treatment)<0.0001, p(Washout)=0.3237, p(Treatment x Washout)=0.8660; Šidák's post hoc tests for treatment effect: ****p<0.0001.

(E) Mean duration of each nose contact; 2-way ANOVA: p(Treatment)<0.0001, p(Washout)=0.9807, p(Treatment x Washout)=0.8922; Šidák's post hoc tests for treatment effect: ****p<0.0001.

(F) Time spent in paw contact; 2-way ANOVA: p(Treatment)<0.0001, p(Washout)=0.1638, p(Treatment x Washout)=0.1465; Šidák's post hoc tests for treatment effect: ****p<0.0001.

(G) Number of paw contacts; 2-way ANOVA: p(Treatment)<0.0001, p(Washout)=0.1793, p(Treatment x Washout)=0.2109; Šidák's post hoc tests for treatment effect: ****p<0.0001.

(H) Mean duration of each paw contact; 2-way ANOVA: p(Treatment)<0.0001, p(Washout)=0.7906, p(Treatment x Washout)=0.6926; Šidák's post hoc tests for treatment effect: ****p<0.0001.

(I) Number of followings; 2-way ANOVA: p(Treatment)<0.0001, p(Washout)=0.7697, p(Treatment x Washout)=0.2517; Šidák's post hoc tests for treatment effect: ***p<0.001, *p<0.05.

(J-N) Motor stereotypies (n=14 Control; n=15 *p*-Cresol):

(J) Number of head shakes; 2-way ANOVA: p(Treatment)<0.0001, p(Washout)=0.2185, p(Treatment x Washout)>0.9999; Šidák's post hoc tests for treatment effect: **p<0.01.

(K) Number of circling events; 2-way ANOVA: p(Treatment)=0.0051, p(Washout)=0.2605, p(Treatment x Washout)=0.5796; Šidák's post hoc tests for treatment effect: *p<0.05 for pre-washout groups, p>0.05 for post-washout groups.

(L) Number of rearings; 2-way ANOVA: p(Treatment)=0.8820, p(Washout)=0.0188, p(Treatment x Washout)=0.7823; Šidák's post hoc tests for treatment effect: p>0.05.

(M) Time spent in grooming; 2-way ANOVA: p(Treatment)=0.2308, p(Washout)<0.0001, p(Treatment x Washout)=0.5929; Šidák's post hoc tests for treatment effect: p>0.05.

(N) Time spent in digging; 2-way ANOVA: p(Treatment)=0.9763, p(Washout)=0.8304, p(Treatment x Washout)=0.5579; Šidák's post hoc tests for treatment effect: p>0.05.

(O) Y-maze spontaneous alternations test: percentage of same-arm returns (n=15 Control, n=14 *p*-Cresol): 2-way ANOVA: p(Treatment)=0.0007, p(Washout)=0.0120, p(Treatment x Washout)=0.8179; Šidák's post hoc tests for treatment effect: *p<0.05.

(P) PCA plots of behavioral scores recorded in the dyadic social interaction test and direct monitoring of motor stereotypies (Fig. S3B-N); ellipses of the 95% confidence intervals are indicated for each group (n=14 Control; n=15 *p*-Cresol).

(B-O) Data are presented as dot-plots featuring means ± SD.

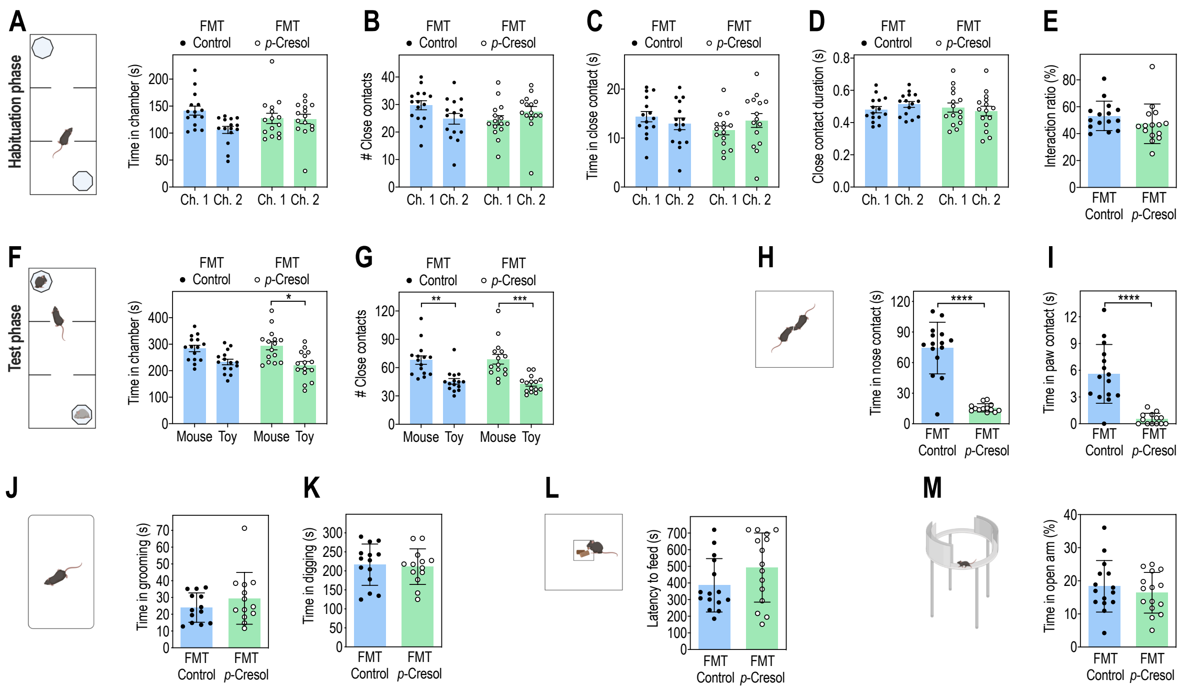

Fig. S4. Additional behavioral parameters for the 3-chamber, dyadic social interactions, motor stereotypies tests and anxiety in FMT^{Control} and FMT$^{p\text{-}Cresol}$ mice (relative to Fig. 4)

(A-E) Additional parameters during the habituation phase in the 3-chamber test post-FMT^{Control} and FMT$^{p\text{-}Cresol}$ (n=15/group):

(A) Time spent in each chamber; 2-way ANOVA: p(FMT)=0.2314, p(Chamber)=0.1231, p(FMT x Chamber)=0.1453; Šidák's post hoc tests for chamber effect: p>0.05.

(B) Number of close contacts with the empty cage in each chamber; 2-way ANOVA: p(FMT)=0.3182, p(Chamber)=0.6725, p(FMT x Chamber)=0.0658; Šidák's post hoc tests for chamber effect: p>0.05.

(C) Time in close contact with the empty cage in each chamber; 2-way ANOVA: p(FMT)=0.4025, p(Chamber)=0.795, p(FMT x Chamber)=0.1127; Šidák's post hoc tests for chamber effect: p>0.05.

(D) Mean duration of each close contact with the empty cage in each chamber; 2-way ANOVA: p(FMT)=0.6674, p(Chamber)=0.2078, p(FMT x Chamber)=0.281; Šidák's post hoc tests for chamber effect: p>0.05.

(E) Interaction ratio (percentage of time spent exploring the wire cage in Chamber 1 relative to the total time spent exploring both wire cages in Chamber 1 and 2); Mann-Whitney U-test: p=0.081.

(F, G) Additional parameters during the test phase in the 3-chamber test post-FMT^{Control} and FMT$^{p\text{-}Cresol}$ (n=13/group):

(F) Time spent in each chamber containing the mouse interactor or the toy mouse; 2-way ANOVA: p(FMT)=0.8256, p(Preference)<0.002, p(FMT x Preference)=0.5436; Šidák's post hoc tests for Mouse vs. Toy preference: p>0.05 for FMT^{Control} group, *p<0.05 for FMT$^{p\text{-}Cresol}$ group.

(G) Number of close contacts with the mouse interactor or the toy mouse; 2-way ANOVA: p(FMT)=0.7887, p(Preference)<0.0001, p(FMT x Preference)=0.6501; Šidák's post hoc tests for Mouse vs. Toy preference: **p<0.01, ***p<0.001.

(H, I) Dyadic social interaction test (n=15 animals/group):

(H) Total time spent in nose contact; Mann-Whitney U-test: ****p<0.0001

(I) Total time spent in paw contact; Mann-Whitney U-test: ****p<0.0001

(J, K) Motor stereotypies (n= 14 $FMT^{Control}$, n=13 $FMT^{\textit{p}\text{-Cresol}}$):

(J) Time spent in grooming; Mann-Whitney U-test: p=0.3358.

(K) Time spent in digging; Mann-Whitney U-test: p=0.5502.

(L) NSF test (n=15 animals/group): latency to first bite; Mann-Whitney U-test: p=0.164.

(M) Zero-maze test (n=15 animals/group): percentage of time spent in open arm; Mann-Whitney U-test: p=0.653.

Data are presented as dot-plots featuring means ± SD.

A

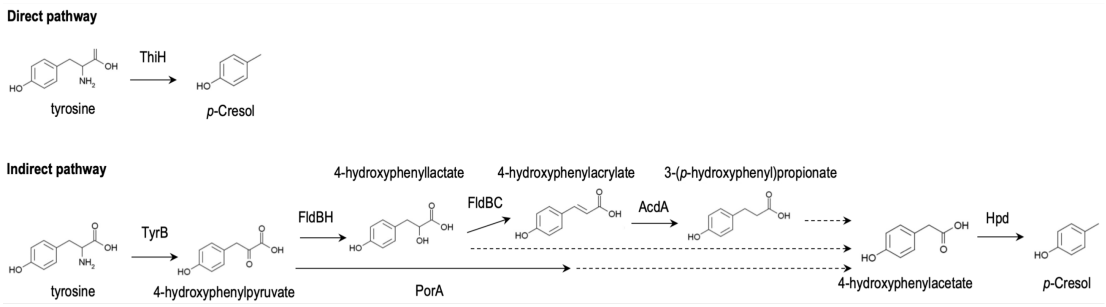

B

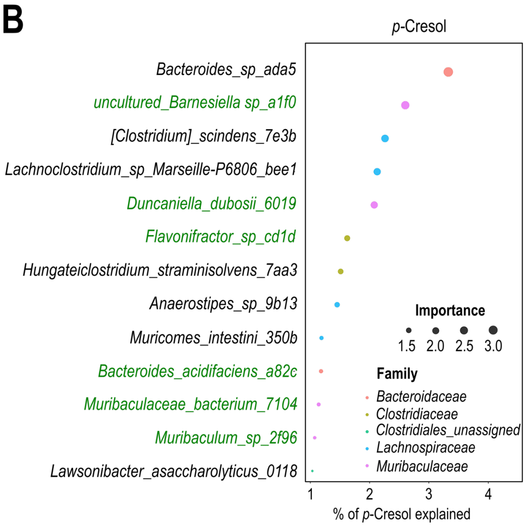

Fig. S5. *p*-Cresol microbial biosynthetic pathways (relative to Fig. 5)

(A) Metabolic pathways from tyrosine to *p*-Cresol. The direct pathway uses thiazole synthase H (ThiH), while the indirect pathway uses several intermediate enzymes: tyrosine aminotransferase B (TyrB), phenyllactate dehydrogenase (FldH), phenyllactate dehydratase (FldBC), acyl-CoA dehydrogenase (AcdA), hydroxyarylic acid decarboxylase (Had), pyruvate-ferredoxin oxidoreductase A (PorA) and hydroxyphenylacetate decarboxylase (Hpd). Dashed lines indicate enzymes not yet identified/characterized. Adapted from [28].

(B) Microbial composition prediction of fecal *p*-Cresol in FMT^{Control} and FMT$^{p\text{-}Cresol}$ mice (n=8 FMT^{Control}, n=12 FMT$^{p\text{-}Cresol}$). ASV best predicting fecal *p*-Cresol as identified by Random Forest analysis. Only ASV contributing >1% accuracy in fecal *p*-Cresol concentration prediction are presented. ASV related to ASV/species increased in FMT$^{p\text{-}Cresol}$ mice as determined by ANCOM analysis are labeled in green.

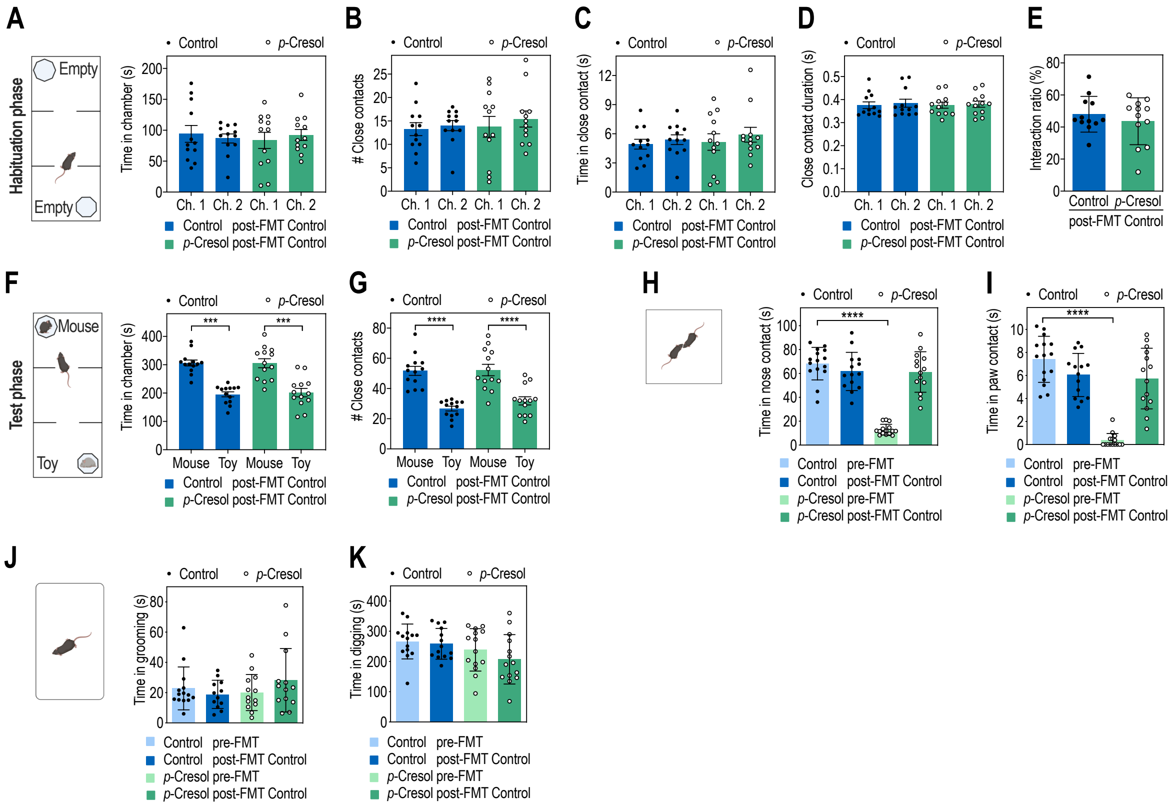

Fig. S6. FMT experiments from control donors (FMT^{Control}) to Control or *p*-Cresol-treated recipient mice: additional behavioral parameters in the 3-chamber, dyadic social interactions and stereotypies tests (relative to Fig. 6)

(A-E) Additional parameters during the habituation phase in the 3-chamber test post-FMT^{Control} (n=13/group):

(A) Time spent in each chamber; 2-way ANOVA: p(Treatment)=0.8296, p(Chamber)=0.9514, p(Treatment x Chamber)=0.4749; Šidák's post hoc tests for chamber preference effect: p>0.05.

(B) Number of close contacts with the empty cage in each chamber; 2-way ANOVA: p(Treatment)=0.6456, p(Chamber)=0.2947, p(Treatment x Chamber)=0.6878; Šidák's post hoc tests for chamber preference effect: p>0.05.

(C) Time in close contact with the empty cage in each chamber; 2-way ANOVA: p(Treatment)=0.6728, p(Chamber)=0.1662, p(Treatment x Chamber)=0.7417; Šidák's post hoc tests for chamber preference effect: p>0.05.

(D) Mean duration of each close contact with the empty cage in each chamber; 2-way ANOVA: p(Treatment)=0.9252, p(Chamber)=0.2163, p(Treatment x Chamber)=0.7061; Šidák's post hoc tests for chamber preference effect: p>0.05.

(E) Interaction ratio (percentage of time spent exploring the wire cage in Chamber 1 relative to the total time spent exploring both wire cages in Chamber 1 and 2); Mann-Whitney U-test: $p=0.755$.

(F,G) Additional parameters during the test phase in the 3-chamber test post-FMT^{Control} (n=13/group):

(F) Time spent in each chamber containing the mouse interactor or the toy mouse; 2-way ANOVA: p(Treatment)=0.7588, p(Preference) <0.0001, p(Treatment x Preference)=0.8356; Šidák's post hoc tests for Mouse vs. Toy preference: ***$p<0.001$.

(G) Number of close contacts with the mouse interactor or the toy mouse; 2-way ANOVA: p(Treatment)=0.3399, p(Preference)<0.0001, p(Treatment x Preference)=0.4225; Šidák's post hoc tests for Mouse vs. Toy preference: ****$p<0.0001$.

(H, I) Additional parameters in the dyadic social interaction test pre- and post-FMT^{Control} (n=15/group pre-FMT, n=14/group post-FMT):

(H) Time spent in nose contact; 2-way ANOVA: p(Treatment)<0.0001, p(FMT^{Control})<0.0001, p(Treatment x FMT^{Control}) <0.0001; Šidák's post hoc tests for treatment effect: ****$p<0.0001$ for pre-FMT^{Control} groups, $p>0.05$ for post-FMT^{Control} groups.

(I) Time spent in paw contact; 2-way ANOVA: p(Treatment)<0.0001, p(FMT^{Control})=0.0002, p(Treatment x FMT^{Control})<0.0001; Šidák's post hoc tests for treatment effect: ****$p<0.0001$ for pre-FMT^{Control} groups, $p>0.05$ for post-FMT^{Control} groups.

(J, K) Additional parameters in the motor stereotypies test pre- and post-FMT^{Control} (n=14/group pre-FMT, n=14/group post-FMT):

(J) Time spent in grooming; 2-way ANOVA: p(Treatment)=0.7605, p(FMT^{Control})=0.2801, p(Treatment x FMT^{Control})=0.9682; Šidák's post hoc tests for treatment effect: $p>0.05$.

(K) Time spent in digging; 2-way ANOVA: p(Treatment)=0.0295, p(FMT^{Control})=0.2740, p(Treatment x FMT^{Control})=0.5058; Šidák's post hoc tests for treatment effect: $p>0.05$.

Data are presented as dot-plots featuring means ± SD.

SUPPLEMENTARY TABLES

Tab. S1. ANCOM analysis: significant microbial features (from ASV to phylum) discriminating *p*-Cresol from Control microbiota (relative to Fig. 3)

Taxonomic information regarding the identified bacterial features, corresponding centered log ratio (CLR), Wald's test W statistic as determined by ANCOM analysis of bacterial composition based on 16S rRNA sequencing are indicated. ASV, amplicon sequence variant (n=30 Control, n=30 *p*-Cresol).

Available as Excel file, Additional file 2.

Tab. S2. ANCOM analysis: significant microbial features (from ASV to phylum) discriminating $FMT^{Control}$ from $FMT^{p\text{-}Cresol}$ mice, 3 weeks post-FMT (relative to Fig. 5)

Taxonomic information regarding the identified bacterial features, corresponding centered log ratio (CLR), Wald's test W statistic as determined by ANCOM analysis of bacterial composition based on 16S rRNA sequencing are indicated. ASV, amplicon sequence variant (n=15 $FMT^{Control}$, n=15 $FMT^{p\text{-}Cresol}$).

Available as Excel file, Additional file 3.

Tab. S3. Output of blast sequence analysis for ThiH, Tyr and HpdA/B/C enzymes involved in *p*-Cresol synthesis (relative to Tab. 1)

Available as Excel file, Additional file 4.

Tab. S4. Details on ASV sequences and taxonomic affiliation. Name and sequence of each of the ASV identified in the dataset, as well as the taxon identity (txid) and homology scores (% of identity, bitscore, e-value) for the nearest homology match.

Available as Excel file, Additional file 5.

Tab. S5: Recapitulation of the behavioral tests performed in the course of the study (✓ assessed, × non-assessed).

Behavioral dimension	Test	*p*-Cresol<br>Fig. 1, Fig. S2	*p-Cresol* washout<br>Fig. S3	FMT$^{p\text{-}Cresol}$ FMT^{Control}<br>Fig. 4, Fig. S4	FMT^{Control}<br>Fig. 6, Fig. S6
**Social behavior**	Dyadic social interaction	✓	✓	✓	✓
	3-chamber test	✓	×	✓	✓
**Stereotypies**	Motor stereotypies	✓	✓	✓	✓
	Marble burying test	✓	×	×	×
	Y-maze	✓	✓	×	×
**Activity**	Actimetry	✓	×	×	×
**Anxiety**	Openfield	✓	×	×	×
	Novelty suppressed feeding	✓	×	✓	×
	Zero-maze	✓	×	✓	×
**Cognition**	Novel object recognition	✓	×	×	×

Tab. S6. Compilation of ANOVA statistics for behavioral data analyses and PERMANOVA statistics for 16S rRNA gene sequencing-based richness and diversity analyses

Available as Excel file, Additional file 6.
